# A pilot study to improve pain phenotyping in head and neck cancer patients

**DOI:** 10.3389/fpain.2023.1146667

**Published:** 2023-05-11

**Authors:** Yi Ye, Diovana de Melo Cardoso, Giseli Mitsuy Kayahara, Daniel Galera Bernabé

**Affiliations:** ^1^ Translational Research Center, New York University College of Dentistry, New York, NY, United States; ^2^ Pain Research Center, Department of Molecular Pathobiology, New York University College of Dentistry, New York, NY, United States; ^3^ Oral Oncology Center, São Paulo State University (UNESP), School of Dentistry, Araçatuba, São Paulo, Brazil

**Keywords:** head and neck cancer, oral cancer, oropharyngeal cancer, neuropathic pain, orofacial pain

## Abstract

Pain associated with head and neck cancer (HNC) is difficult to manage and reduces quality of life. It has been increasingly recognized that HNC patients exhibit a wide range of pain symptoms. Here we developed an orofacial pain assessment questionnaire and conducted a pilot study to improve pain phenotyping in HNC patients at the diagnosis. The questionnaire captures the following pain characteristics: pain intensity, location, quality, duration, and frequency; the impact of pain on daily activities; changes in smell and food sensitivities. Twenty-five HNC patients completed the questionnaire. 88% patients reported pain at the site of tumor; 36% reported multiple pain sites. All patients with pain reported at least one neuropathic pain (NP) descriptor, 54.5% reported at least two NP descriptors. The most common descriptors were “burning” and “pins and needles”. Most patients reported increased pain to sour or hot/spicy food/drinks, and to food with coarse/hard textures. Patients exhibited impaired oral function, especially chewing, talking, mouth/jaw opening, and eating. Tumor progression has a significant impact on pain. Nodal metastasis is linked to pain at multiple body sites. Patients with advanced tumor staging experience greater pain at the primary tumor site, when exposed to hot or spicy food/drinks or food with hard/coarse texture, or when eating or chewing. We conclude that HNC patients experience a wide range of pain symptoms with altered mechanical, chemical, and temperature sensation. Improved phenotyping and stratification of pain in HNC patients will help address the underlying etiology, which may enable personalized therapeutic approaches in the future.

## 1. Introduction

Pain affects more than 70% of head and neck cancer (HNC) survivors. Chronic pain resulted from cancer or cancer treatments such as surgery, radiation, and chemotherapies or in combination, is particularly challenging as patients suffer from multiple oral complications such as orofacial pain, changes in taste, and oral dysfunction (1, 2). These oral complications significantly comprise patient's quality of life socially and nutritionally. Preexisting oral complications and associated comorbidities before treatment predict the severity of post treatment symptoms (2, 3). Improved phenotyping and stratification before treatment provide opportunities for a better understanding of etiology underlying post-treatment oral complications and might allow a precision approach to treatment selection.

In this pilot study, we designed a simple orofacial pain questionnaire (OFPQ) with the aim to simultaneously capture pain quality, intensity, and frequency at each painful body site, spontaneous pain as well as evoked pain, the extent of pain affecting daily function/activities, and finally, the sensory and taste disturbance in the oral cavity due to the disease. We found that as reported previously, HNC patients experience both spontaneous and evoked oral pain at the site of tumor (4–6). Surprisingly, a subset of patients experience pain away from the site of tumor (*i.e*., referred pain), some experience pain at multiple locations. For the first time, we report that a subset of HNC patients develop increased pain sensitivities to sour and hot/spicy food as well as food with hard/coarse texture, suggesting chemical, thermal, and mechanical hypersensitivities at the oral cavity. There was a strong correlation between nodal metastasis and referred pain. Patients with advanced tumor staging experience greater pain at the primary tumor site, when exposed to hot or spicy food/drinks or food with hard/coarse texture, or when eating or chewing. Pain intensity at the tumor site has a significant impact on the total sensory scores as well as the total function scores. The next step is to refine the questionnaire and validate our study in a bigger cohort.

## 2. Patients and methods

### 2.1. The orofacial neuropathic pain questionnaire

The validated UCSF oral cancer pain questionnaire (UCSFOCPQ) has been used extensively to characterize intensity, sharpness, and aching nature of spontaneous and evoked pain, touch sensitivities, as well as oral function as a result of pain (4, 7) focusing on the site of tumor. To understand whether HNC patients experience other pain symptoms that are not captured in UCSFOCPQ, we constructed the OFPQ that incorporates elements from UCSFOCPQ, the Neuropathic Pain Symptom inventory (NPSI) (8), as well as the Brief Pain Inventory (BPI) (9). In addition, we asked other questions that are specific to the oral function, such as changes in food taste/sensitivities. The OFPQ contains four parts (See Supplementary Materials). First, patients were instructed to points out each painful body parts and rate the respective pain intensity. In the second part patients were instructed to rate their pain using neuropathic pain descriptors like burning, pins and needles, pricking, shooting, painful cold, pinching, electric shock, tingling, and numbness for each pain site. The pain frequency and intensity and whether the pain is the stimulus dependent (provoked or continuous) for each pain site is also recorded. In the third part, the patient is encouraged to report how pain affects their physiological functions and activities daily. In the last part of the questionnaire, the occurrence and intensity of taste/smell/oral sensory symptoms (i.e., pain evoked by food) are explored. Measurements of pain intensity, impairment of daily activities and sensory symptoms were made using a 0–10 numeric rating scale (NRS) with 0 being no pain (or no impact) and 10 being the worse imaginable pain (or the highest imaginable impact).

### 2.2. Patients

A single-center, prospective study was carried out to assess orofacial pain in patients with HNC. The study was conducted from July 2021 to July 2022. The study complied with the guidelines for human studies and was conducted in accordance with the “World Medical Association Declaration of Helsinki”. The study was approved by the Committee of Ethics for Research Human Studies of the Sao Paulo State University (UNESP), School of Dentistry, Araçatuba, Sao Paulo, Brazil and informed consent was obtained from all participants. Only de-identified information was shared for data analysis. Twenty-five patients with histology proven head and neck squamous cell carcinoma (HNSCC) who referred for oncological treatment at the Oral Oncology Center, UNESP, School of Dentistry, Araçatuba, São Paulo, Brazil, were enrolled in this study. Inclusion criteria were patients over 18 years of age; histopathological diagnosis of HNSCC; and primary tumor located in the oral cavity, oropharynx, or larynx. Exclusion criteria were patients reporting preexisting pain related with other non-oncological diseases; with previous oncological treatment; or inability to answer the orofacial neuropathic pain questionnaire. A healthcare professional explained each questionnaire items to patients and all patients were interviewed before starting cancer treatment.

### 2.3. Statistical analysis

GraphPad *Prism 9 was* used to perform the statistical analyses. Standard parametric (Student's *t*-test, one-way ANOVA, two-way ANOVA) or nonparametric (Mann-Whitney U-test, Kruskal-Wallis) tests were used, depending on normality. Chi-square or Fisher's exact test was used to determine the difference in distribution between groups. Results were presented as mean ± standard deviation (SD), mean ± standard error of the mean (SEM), or mean (range), wherever appropriate. Significance level was set at *P < 0.05, **P < 0.01, ***P < 0.001.

## 3. Results

### 3.1. Patient demographics

A total of 25 HNSCC patients with an age range of 42–78 years (mean = 61.6 years) completed the questionnaire at the diagnosis visit. Demographic data of the entire patient cohort are shown in Table 1. Most subjects were male (19, 76%) or had SCC located in the oral cavity (18, 72%). The most common primary site of the tumor was tongue (24%), floor of the mouth (19%), followed by the alveolar ridge (11.5%). Early and advanced disease was well represented (Clinical stage I–II, *n* = 12; 48% and stage III–IV *n* = 13; 52%). 9 (36%) patients had neck nodal invasion. None of the patients had remote metastasis.

**Table 1:** Characteristics of patients who completed the orofacial pain questionnaire (OFPQ).

Variables	*N* (%) 25 (100%)
Age (years) mean (SD), range	61.6 (9.08); 42–78
Male	19 (76%)
Female	8 (24%)
Cancer clinical stage	
I	4 (16%)
II	8 (32%)
III	4 (16%)
IV	9 (36%)
T staging	
1	5 (20%)
2	9 (36%)
3	7 (28%)
4	4 (16%)
Nodal metastasis	
Yes	9 (36%)
No	16 (64%)
Tumor site	
**Oral**	18 (72%)
Floor of mouth	5
Tongue	6
Alveolar ridge	3
Lower lip	2
Cheek	1
Buccal mucosa	1
**Oropharynx**	6 (24%)^a^
Tonsillar pillar	2
Soft palate	2
Palatine tonsil	2
Base of the tongue	2
**Larynx**	1 (4%)

^a^ One patient has primary tumors at the soft palate, palatine tonsil, and the base of the tongue.

### 3.2. Prevalence, quality, and severity of pain

**Figure 1:**
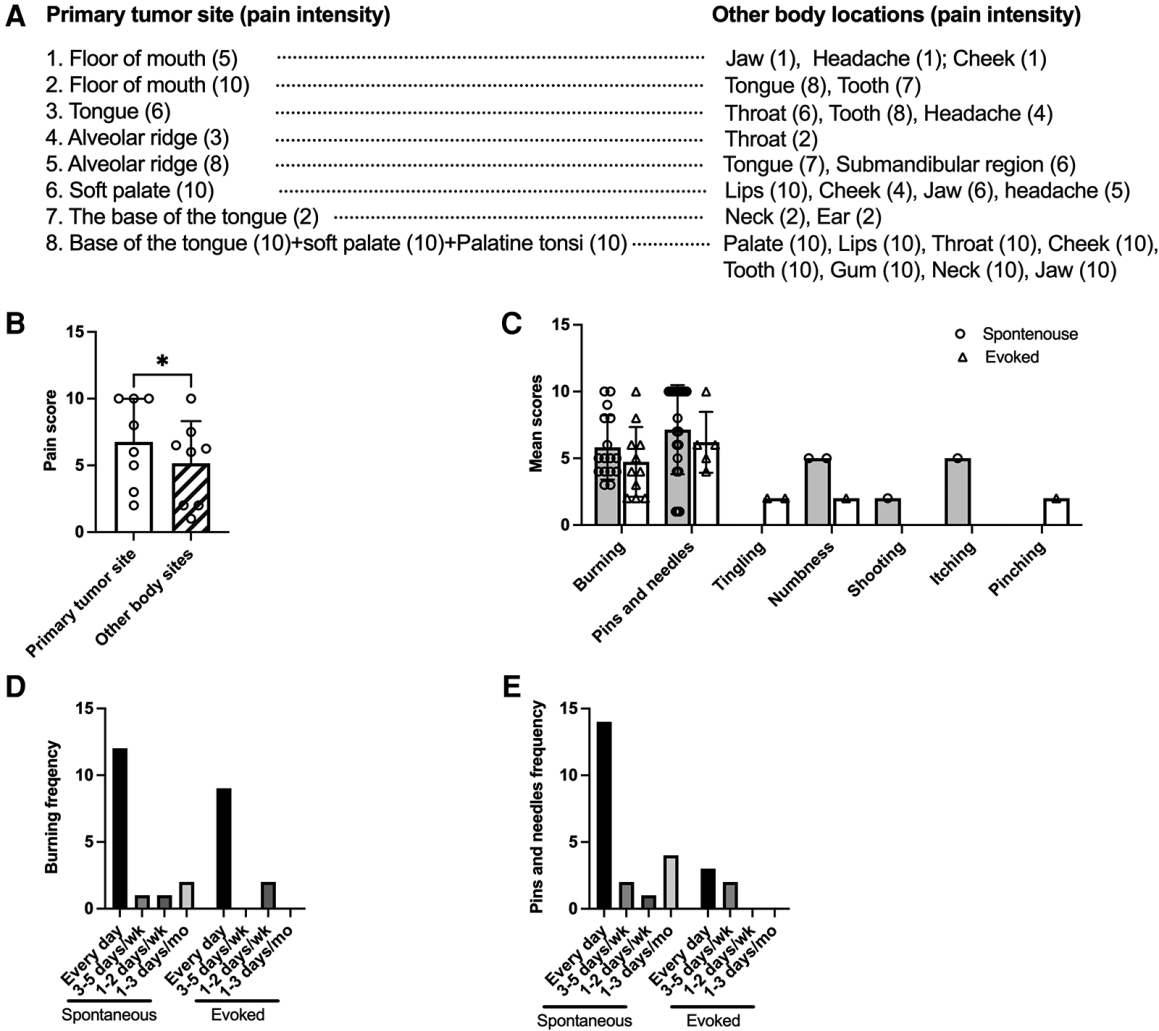
Pain at multiple sites, spontaneous and evoked NP descriptors and their frequency. (**A**) Eight patients reported pain at the primary tumor site as well as at other body regions. The numbers in the brackets indicate pain scores for each body location. (**B**) The comparison of pain scores at the primary tumor site and the mean pain score of the other body sites. Paired *t*-test. (**C**) The mean score and number of patients used neuropathic pain descriptors are shown. Most patients reported both spontaneous (*n* = 16) and evoked (*n* = 21) burning sensations. Eleven patients reported spontaneous pins and needles, five reported evoked pins and needles. (**D**) Frequency for spontaneous burning and evoked burning in the past month. (**E**) Frequency for spontaneous and evoked pins and needles in the past month.

Twenty-two (88%) HNC patients reported pain at the site of tumor. Eight of them (36%) reported pain in other orofacial pain regions in addition to the site of tumor, with a mean of 4 sites per patient (Table 2 and Figure 1A). The site of the tumor is the most painful site for 87.5% of patients who reported pain at multiple locations (Table 2 and Figures 1A,B). The mean pain intensity at the site of tumor on a 0- to 10-point numerical rating scale was 5.4 (Table 2). There is no difference between male vs. female in pain intensity at the tumor site (Table 2). All patients reported at least one NP descriptor, 54.5% reported at least two NP descriptors (Table 2). The most common NP descriptor was “burning” (frequency = 16 for spontaneous burning; frequency = 21 for evoked burning) followed by “pins and needles” (frequency = 11 for spontaneous pins and needles; frequency = 6 for evoked pins and needles). Tingling (*n* = 2), numbness (*n* = 1), itching (*n* = 1), pinching (*n* = 1), shooting (*n* = 1) are not common among the 22 patients who reported pain. For patients who reported “burning” and “pins and needles”, most patients experience them on daily basis (Figures 1C–E). Six patients were using analgesics for their pain management when the questionnaire was applied (data not shown).

**Table 2:** Neuropathic pain characteristics.

Variables	*N*	Mean pain intensity at the primary tumor site (SD)	*P*-value
Pain free	3		
With pain	22	5.409 (2.482)	
Male	19	4.368 (2.449)	0.243
Female	6	6.0 (4.05)	
Pain at one site	14	4.643 (1.598)	0.053
Pain at >1 site	8	6.75 (3.24)	
Mean pain score at the other body part		5.15 (3.16)	
Early stage (I–II)	12	3.25 (2.49)	0.04*
Advanced stage (III–IV)	13	5.56 (2.81)	
T stage 1–2	14	3.357 (2.27)	0.004**
T stage 3–4	11	6.545 (2.77)	
N0	9	5.889 (3.371)	0.15
N+	16	4.125 (2.553)	
Used 1 NP descriptors	10	4.214 (3.286)	0.37
Used 2 or more NP descriptors	12	5.25 (2.379)	

*p<0.05; **p<0.01.

### 3.3. Sensitivities to food and smell

**Figure 2:**
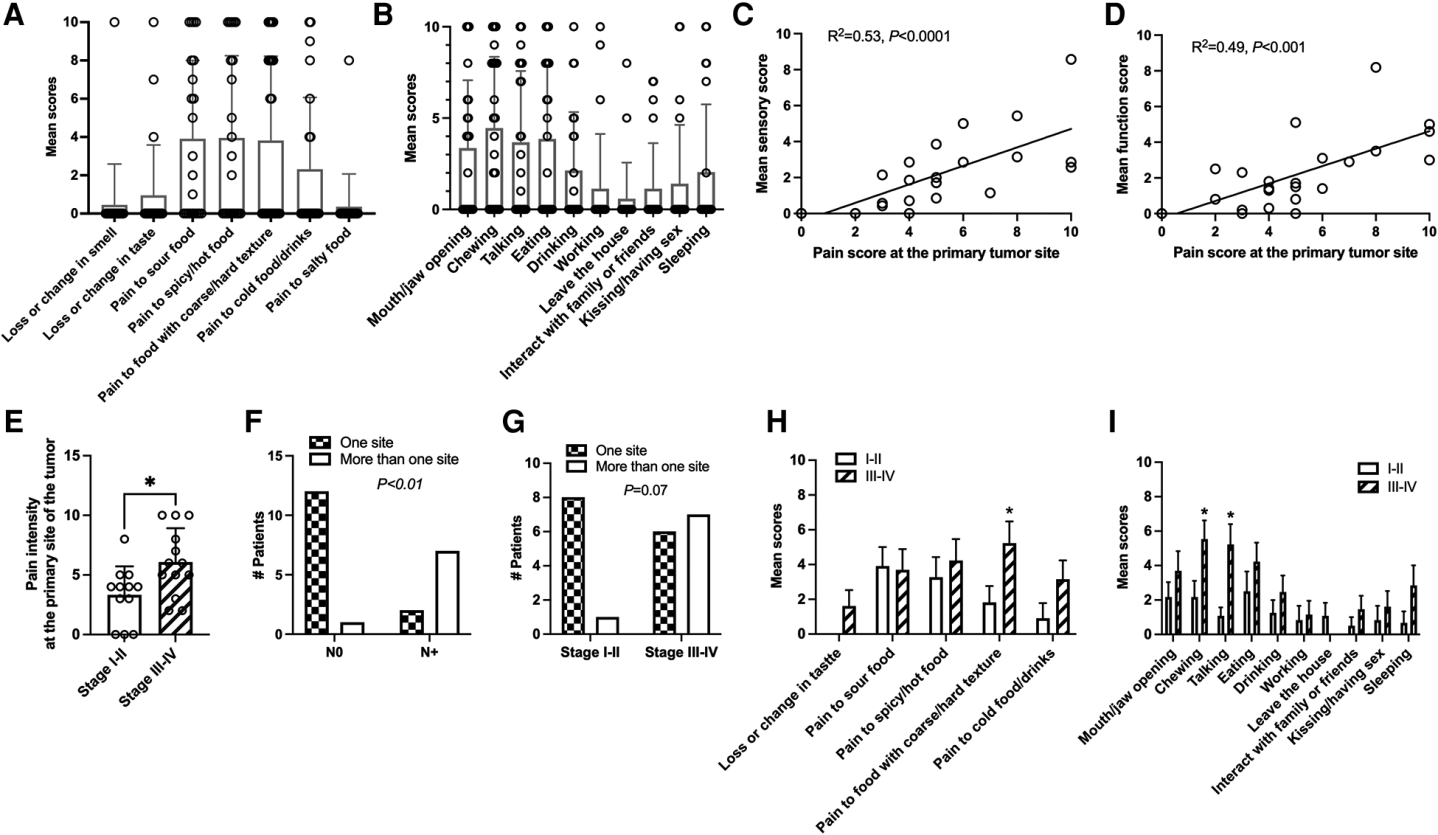
Food sensitivities and functional impact of pain get worse with disease progression. (**A**) A big proportion of patients developed pain to sour (*n* = 14), spicy or hot food/drinks (*n* = 13), or food with hard/coarse texture (*n* = 11). (**B**) Patients reported a big impact of pain on oral functioning such as mouth/jaw opening (*n* = 12), chewing (*n *= 15), talking (*n* = 13), eating (*n* = 12) and drinking (*n* = 9). (**C**) The mean total sensory score correlated strongly with pain intensity at the primary tumor site for each patient. Simple linear regression. (**D**) The mean total function score correlated strongly with pain intensity at the primary tumor site. Simple linear regression. (**E**) Pain intensity at the site of the tumor is significantly greater in patients with advanced cancer (clinical stage III–IV) compared to patients with early cancer (clinical stage I–II). Student's *t*-test. (**F**) Patients with nodal metastasis are more likely to have pain at multiple body sites. (**G**) There is a trend that patients with advanced cancer (clinical stage II-IV) are more likely to develop pain at multiple body sites. Chi-Square. (**H**) Pain to food with coarse/hard texture is greater in patients with advanced tumors (clinical stage III–IV) compared to patients with early-stage tumors (clinical stage I–II). Chi-Square. (**I**) Pain has a greater impact for chewing and talking in patients with advanced tumors (clinical stage III–IV) compared to patients with early-stage tumors (clinical stage I–II). Student's *t*-test.

Patients exhibited profound sensory alteration in response to food (Figure 2). More than half of patients reported pain to sour food (*n* = 14), to spicy/hot food or drinks (*n* = 13), and pain to food with coarse/hard textures (*n* = 11) more than usual (Figure 2A). A smaller percentage of patients also reported loss or change in smell, pain to cold food/drinks, and to salty food. The mean total score of food and smell sensitivities correlated strongly with the mean pain intensity at the primary tumor site (*R*^2 ^= 0.53, *P* < 0.0001; Figure 2C).

### 3.4. The impact of pain on daily function

Like what has previously reported using a validated UCSFOCPQ (4, 5, 7), patients exhibited significantly impaired oral function due to pain, especially chewing (*n* = 15), mouth/jaw opening (*n* = 12), talking (*n* = 13), eating (*n* = 12), and to a lesser degree drinking (*n* = 9, Figure 2B). A smaller percentage of patients reported that pain affects their ability to walk, leaving the house, having sex/kissing, interacting with family members and friends, and sleeping (Figure 2B). The mean total score of daily function correlated strongly with the pain intensity at the site of the tumor (*R*^2 ^= 0.49, *P* < 0.001, Figure 2D).

### 3.5. Association between tumor progression and pain

We found that clinical staging or tumor T staging have significant effects on pain severity at the site of tumor. The general pain mean scores are significantly higher in patients with advanced clinical stage (III-IV), compared to those with early-stage (I and II) (Table 2 and Figure 2E). Similarly, patients with T3 and T4 stages have significantly higher pain intensity at the site of the tumor compared to T1 and T2 patients (Table 2). Nodal metastasis was significantly associated with pain at multiple sites (Figure 2F), whereas patients with advanced tumor staging exhibited a tendency with pain at multiple sites (Figure 2G). HNC patients with advanced stage (III-IV) also reported higher pain when eating food with coarse/hard texture and when chewing or talking (Figures 2H,I). No difference in pain intensity at the site of tumor (Table 2), food/smell sensitivity ratings (data not shown), or function (data not shown) was found between male vs. female, nodal metastasis vs. no nodal metastasis (Table 2), or among patients with pain at one site vs. multiple sites (Table 2), or between patients used one NP descriptor or used two or more NP descriptors (Table 2). There is no correlation between age and pain intensity at the primary tumor site (data not shown).

## 4. Discussion

The purpose of this pilot to study is to determine whether we can capture thermal and chemical hypersensitivities, referred pain, as well as pain quality that are suggestive of neuropathic nature, which are not commonly included in existing orofacial pain questionnaires. Orofacial pain is unique in that the orofacial region not only has rich blood and nerve innervation in a confined space, but it also involves multiple specialized organs such as mouth, tongue, lips, tooth, jaw that serve critical functions such as eating, talking, drinking, which have significantly impact on patients' social life and nutritional status. Orofacial pain associated with HNC and its treatment such as surgery, chemotherapy, and or radiotherapy reduce quality of life and survival. It has been increasingly recognized that pre-existing pain condition could influence pain following cancer treatment, especially pre-existing NP which is more distressing than nociceptive pain, with a greater impact on life and health (10, 11). There are several questionnaires designed to screen and assess NP including PainDETECT (10), Leeds Assessment of Neuropathic Symptoms and Signs (LANSS) (12), DN4 (13) and NPSI (8). However, none of these are specifically designed for orofacial pain. As there is no indicative pain biomarkers or readily available diagnostic tools, a conclusive diagnosis of NP is difficult (14). Therefore, in this OFPQ we included NP descriptors to better characterize pain suffered by patients with HNC.

The major finding of the study is that orofacial pain associated with HNC is heterogeneous; patients exhibit a combination of symptoms that are unique to them. This new OFPQ validated results from UCSFOCPQ that spontaneous and evoked pain is prevalent in HNC patients at the site of the tumor, which limits oral function. However, a subset of HNC patients exhibit a wide range of symptoms, such as referred pain, burning pain, pins and needles, and pain sensitivities to food at the cancer diagnosis. Patients may describe their pain as pins and needles at one location and stabbing at a different body location. The frequency and duration for different pain subtypes also varies dramatically among patients. The heterogeneity of the pain may reflect differences in anatomy, pathophysiology, and socio-psychological factors among patients.

The most interesting finding is that HNC patients report to have increased pain sensitivities to hot/spicy and sour food as well as food with hard/coarse texture. While mechanical allodynia of the tongue has been reported extensively, chemical, heat, or cold-induced pain are rarely assessed in HNC patients (6, 15). A recent study reported that cancer patients exhibit increased sensitivities to capsaicin at the site of tumor (15). Incorporating food sensitivities represents a convenient way to assess these important sensory modalities unique to the oral region. Increased pain triggered by food as well as taste/smell disturbance are significant contributors of malnutrition, which is a major clinical concern of HNC patients following cancer treatment (1, 2).

The mechanism underlying mechanical, thermal, and chemical hypersensitivities can be attributed to the interaction between the tumor microenvironment and peripheral nerves. The HNC microenvironment is acidic (16); HNC and its stromal cells release soluble mediators and extracellular vesicles which could activate or sensitize receptors that are important for mechano-, thermos, or chemical sensing (6, 17, 18). The capsaicin receptor TRPV1, which can be also activated by mechanical stimulus or protons, is often sensitized in mice models of HNC (19–22). The nerves innervating the orofacial region also express cold/mechanical sensitive ion channels like TRPA1, cold sensitive TRPM8, or acid-sensing channels like (ASICs and P2X) (23, 24), which can be directly or indirectly stimulated by H^+^, ATP (25), lipids (20), growth factors (21, 26), proteases (27–29), or cytokines (30–32) released by the tumor microenvironment.

We found a strong correlation between tumor staging with pain intensity at the tumor site, pain caused by hot/spicy food and by food with coarse/hard texture, as well as limited oral function. Both clinical tumor staging, and T-staging are influenced by tumor size. While some studies found a correlation between tumor size and pain, it should be noted that others found other pathological features, such as nodal metastasis, to be correlated with HNC pain severity (4, 7).

Our study has limitations. First, we instructed patients to report their pain-related symptoms within the past month. Some patients may experience pain longer than one month. Also, a proportion of patients have pain at different body locations, which may not develop at the same time. The questionnaire can be improved by asking patients “How long you have noticed this pain?” for each pain site. Second, the duration for food/taste sensitivities is not recorded by the questionnaire. Understanding the timeline for the start and duration of each symptom may provide a clue regarding the temporal relationship among symptoms. Third, other factors or set of factors associated with pain may have been omitted. We routinely use the Hospital Anxiety and Depression Scale (HADS) and the Pittsburgh Sleep Quality Indenx (PSQI) to investigate symptoms of anxiety and depression and sleep disturbance in HNC patients. These questionnaires can be used in conjunction with the OFPQ to factor in pain-associated comorbidities. As a pilot study, the questionnaire specificity and reliability were not tested as we expect further refinement of the questionnaire may be needed. Lastly, our sample size is very small which is not sufficiently powered to answer a few important questions related to HNC pain. For example, although our study agrees with known epidemiology that HNC affects men three times more than women (33), our study is not powered to detect sex difference in HNC pain (only a trend of increased pain in females) (6, 34). Studies have reported pain to be more severe in women (35–37), or in men (4, 38), or no difference (34). Whether patients with advanced tumor exhibited more NP features is another question that our study is not powered to answer. We plan to expand our cohort following the refinement of the questionnaire and test its specificity and sensitivity.

In summary, this pilot study identified diverse pain features that are unique to patients with HNC. Improved phenotyping and stratification before treatment provide opportunities for a better understanding of etiology underlying post-treatment oral complications, which may enable personalized therapeutic approaches in the future.

## Data Availability

The raw data supporting the conclusions of this article will be made available by the authors, without undue reservation.
