# Supplementary material for: A pilot study to improve pain phenotyping in head and neck cancer patients
Source: Front Pain Res (Lausanne). 2023 May 11;4:1146667. doi: 10.3389/fpain.2023.1146667 (PMC10211332; doi:10.3389/fpain.2023.1146667)
Supplement: Supplementary file 1 [file Datasheet1.docx]

**Orofacial Pain Questionnaire (OFPQ)**

Name: __________________________________________________________________________ Age: ______ Sex: _______

Date: ________________________

1. Mark the locations that you have pain; if you have multiple locations, please rank them from 1 as the location with the most pain. Rank your pain intensity from 1-10 for each location. 10 being most unbearable pain.

☐Tongue 0 1 2 3 4 5 6 7 8 9 10

☐ Lips 0 1 2 3 4 5 6 7 8 9 10

☐ Throat 0 1 2 3 4 5 6 7 8 9 10

☐ Cheek 0 1 2 3 4 5 6 7 8 9 10

☐ Tooth 0 1 2 3 4 5 6 7 8 9 10

☐ Gum 0 1 2 3 4 5 6 7 8 9 10

☐ Neck 0 1 2 3 4 5 6 7 8 9 10

☐ Jaw 0 1 2 3 4 5 6 7 8 9 10

☐ Headaches 0 1 2 3 4 5 6 7 8 9 10

☐ Other:____________________ 0 1 2 3 4 5 6 7 8 9 10

1. **Ongoing Pain & evoked pain**
2. In the past month, do you have pain with any of the following characteristics without moving/using the area such as talking, eating, drinking (spontaneous pain) or with moving/using the area such as talking, eating, drinking (evoked pain) ? Indicate the location and Rank the pain or symptom from 0-10, 10 being most unbearable.

Location ( ) Burning

☐ Spontaneous 0 1 2 3 4 5 6 7 8 9 10

How often? ☐ Every day; ☐ 3-5 days per week; ☐ 1-2 days per week; ☐ 1-3 days per month;

☐ Evoked 0 1 2 3 4 5 6 7 8 9 10

How often? ☐ Every day; ☐ 3-5 days per week; ☐ 1-2 days per week; ☐ 1-3 days per month;

Does the pain linger when you stopped moving/using the area?

☐ Yes ☐ No

For how long? ☐<1 hour; ☐1-3 hours; ☐> 3hours

Location ( ) Painful cold

☐ Spontaneous 0 1 2 3 4 5 6 7 8 9 10

How often? ☐ Every day; ☐ 3-5 days per week; ☐ 1-2 days per week; ☐ 1-3 days per month;

☐ Evoked 0 1 2 3 4 5 6 7 8 9 10

How often? ☐ Every day; ☐ 3-5 days per week; ☐ 1-2 days per week; ☐ 1-3 days per month;

Does the pain linger when you stopped moving/using the area?

☐ Yes ☐ No

For how long? ☐<1 hour; ☐1-3 hours; ☐> 3hours

Location ( ) Electric shocks

☐ Spontaneous 0 1 2 3 4 5 6 7 8 9 10

How often? ☐ Every day; ☐ 3-5 days per week; ☐ 1-2 days per week; ☐ 1-3 days per month;

☐ Evoked 0 1 2 3 4 5 6 7 8 9 10

How often? ☐ Every day; ☐ 3-5 days per week; ☐ 1-2 days per week; ☐ 1-3 days per month;

Does the pain linger when you stopped moving/using the area?

☐ Yes ☐ No

For how long? ☐<1 hour; ☐1-3 hours; ☐> 3hours

Location ( ) Tingling (like ants crawling)

☐ Spontaneous 0 1 2 3 4 5 6 7 8 9 10

How often? ☐ Every day; ☐ 3-5 days per week; ☐ 1-2 days per week; ☐ 1-3 days per month;

☐ Evoked 0 1 2 3 4 5 6 7 8 9 10

How often? ☐ Every day; ☐ 3-5 days per week; ☐ 1-2 days per week; ☐ 1-3 days per month;

Does the pain linger when you stopped moving/using the area?

☐ Yes ☐ No

For how long? ☐<1 hour; ☐1-3 hours; ☐> 3hours

Location ( ) Pins and needles

☐ Spontaneous 0 1 2 3 4 5 6 7 8 9 10

How often? ☐ Every day; ☐ 3-5 days per week; ☐ 1-2 days per week; ☐ 1-3 days per month;

☐ Evoked 0 1 2 3 4 5 6 7 8 9 10

How often? ☐ Every day; ☐ 3-5 days per week; ☐ 1-2 days per week; ☐ 1-3 days per month;

Does the pain linger when you stopped moving/using the area?

☐ Yes ☐ No

For how long? ☐<1 hour; ☐1-3 hours; ☐> 3hours

Location ( ) Pinching

☐ Spontaneous 0 1 2 3 4 5 6 7 8 9 10

How often? ☐ Every day; ☐ 3-5 days per week; ☐ 1-2 days per week; ☐ 1-3 days per month;

☐ Evoked 0 1 2 3 4 5 6 7 8 9 10

How often? ☐ Every day; ☐ 3-5 days per week; ☐ 1-2 days per week; ☐ 1-3 days per month; Does the pain linger when you stopped moving/using the area?

☐ Yes ☐ No

For how long? ☐<1 hour; ☐1-3 hours; ☐> 3hours

Location ( ) Shooting (like knife stabbing)

☐ Spontaneous 0 1 2 3 4 5 6 7 8 9 10

How often? ☐ Every day; ☐ 3-5 days per week; ☐ 1-2 days per week; ☐ 1-3 days per month;

☐ Evoked 0 1 2 3 4 5 6 7 8 9 10

How often? ☐ Every day; ☐ 3-5 days per week; ☐ 1-2 days per week; ☐ 1-3 days per month;

Does the pain linger when you stopped moving/using the area?

☐ Yes ☐ No

For how long? ☐<1 hour; ☐1-3 hours; ☐> 3hours

Location ( ) Numbness

☐ Spontaneous 0 1 2 3 4 5 6 7 8 9 10

How often? ☐ Every day; ☐ 3-5 days per week; ☐ 1-2 days per week; ☐ 1-3 days per month;

☐ Evoked 0 1 2 3 4 5 6 7 8 9 10

How often? ☐ Every day; ☐ 3-5 days per week; ☐ 1-2 days per week; ☐ 1-3 days per month;

Does the numbness linger when you stopped moving/using the area?

☐ Yes ☐ No

For how long? ☐<1 hour; ☐1-3 hours; ☐> 3hours

Location ( ) Itching

☐ Spontaneous 0 1 2 3 4 5 6 7 8 9 10

How often? ☐ Every day; ☐ 3-5 days per week; ☐ 1-2 days per week; ☐ 1-3 days per month;

☐ Evoked 0 1 2 3 4 5 6 7 8 9 10

How often? ☐ Every day; ☐ 3-5 days per week; ☐ 1-2 days per week; ☐ 1-3 days per month;

Does the itching linger when you stopped moving/using the area?

☐ Yes ☐ No

For how long? ☐<1 hour; ☐1-3 hours; ☐> 3hours

1. On a scale of 0-10, how much does the pain affect your daily function of the area (such as mouth/jaw opening, chewing, talking, eating, drinking…). 10 is the biggest impact possible.

☐ mouth/jaw opening 0 1 2 3 4 5 6 7 8 9 10

☐ chewing 0 1 2 3 4 5 6 7 8 9 10

☐ talking 0 1 2 3 4 5 6 7 8 9 10

☐ eating 0 1 2 3 4 5 6 7 8 9 10

☐ drinking 0 1 2 3 4 5 6 7 8 9 10

☐ working 0 1 2 3 4 5 6 7 8 9 10

☐ leave the house 0 1 2 3 4 5 6 7 8 9 10

☐ interact with family or friends 0 1 2 3 4 5 6 7 8 9 10

☐ kissing/having sex 0 1 2 3 4 5 6 7 8 9 10

☐ sleeping 0 1 2 3 4 5 6 7 8 9 10

**3. Do you develop any of the following sensory symptoms in the past month? Rate change/loss in smell, taste, and pain intensity from 0-10 (0-no loss/change/pain, 10-max. loss/change/pain possible)**

☐ Loss or change in smell ☐yes ☐no

To which degree (0-no loss/change, 10 complete loss/change)

0 1 2 3 4 5 6 7 8 9 10

☐ Loss or change in taste ☐yes ☐no

0-no loss/change, 10 complete loss/change

0 1 2 3 4 5 6 7 8 9 10

☐Pain to sour food/drinks (like lemon, vinegars) 0 1 2 3 4 5 6 7 8 9 10

☐ Pain to hot or spicy food/drinks 0 1 2 3 4 5 6 7 8 9 10

☐ Pain to food with coarse/hard texture 0 1 2 3 4 5 6 7 8 9 10

☐ Other_________________________ 0 1 2 3 4 5 6 7 8 9 10
